# To: Noninvasive positive pressure ventilation after extubation: features and outcomes in clinical practice

**DOI:** 10.5935/0103-507X.20160016

**Published:** 2016

**Authors:** Pedro Silva Santos, Antonio M. Esquinas

**Affiliations:** Pulmonology Unit, Centro Hospitalar e Universitário de Coimbra - Hospitais da Universidade de Coimbra - Coimbra, Portugal.; Intensive Care Unit, Hospital Morales Meseguer - Murcia, Spain.

**To the editor,**

We carefully read the article by Yamauchi et al.^(1)^ on using noninvasive positive pressure ventilation (NIPPV) after extubation and congratulate the authors on their study. However, in the last few years, several studies with conflicting results have been published.^(1-6)^

The authors presented the results from a retrospective observational study. Data were collected over a seven-month period to describe post-extubation NIPPV use in an intensive care unit (ICU) in clinical practice and to identify factors associated with NIPPV failure.^(1)^ Their study included 174 patients who received NIPPV after extubation. The failure rate of NIPPV was high (34%). Another interesting result was that patients with inspiratory positive airway pressure (IPAP) ≥ 13.5cmH2O on the last day of NIPPV support were three times more likely to experience NIPPV failure than patients who had lower IPAP. In this study, the authors concluded that the final NIPPV parameters, length of ICU stay and mortality rate were higher in the NIPPV failure group.

There were, however, some limitations to the study that should be considered.

First, because the study was performed at a single university hospital in Brazil, we propose to increase the number of study centers to make a future study more representative of ICUs in Brazil.

Second, we should consider that the composition of the study population may influence the results. For example, there is a low proportion of patients with chronic respiratory disease, including chronic obstructive pulmonary disease, and such patients usually benefit from NIPPV.^(2)^ In contrast, several studies have shown that the benefit from NIPPV in patients with acute hypoxemic respiratory failure is less clear.^(2,4)^ This finding may explain why, in a group of patients with multiple pathologies, especially in observational studies, there is a high failure rate of NIPPV, as well as why it is difficult to establish a cutoff value for NIPPV failure. Although the study by Rana et al.^(3)^ did not find any association between IPAP levels and NIPPV outcomes, the results presented above represent new challenges for future studies that will investigate the parameters of IPAP and NIPPV to improve clinical practice and decrease ICU mortality.

Further prospective clinical trials are needed to consolidate these results.

Pedro Silva Santos Pulmonology Unit, Centro Hospitalar e Universitário de
Coimbra - Hospitais da Universidade de Coimbra - Coimbra, Portugal.Antonio M. Esquinas Intensive Care Unit, Hospital Morales Meseguer -
Murcia, Spain.

## References

[1] Yamauchi LY, Figueiroa M, Silveira LT, Travaglia TC, Bernardes S, Fu C (2015). Noninvasive positive pressure ventilation after extubation:
features and outcomes in clinical practice. Rev Bras Ter Intensiva.

[2] Esteban A, Frutos-Vivar F, Ferguson ND, Arabi Y, Apezteguía C, González M (2004). Noninvasive positive-pressure ventilation for respiratory failure
after extubation. N Engl J Med.

[3] Rana S, Jenad H, Gay PC, Buck CF, Hubmayr RD, Gajic O (2006). Failure of noninvasive ventilation in patients with acute lung
injury: observational cohort study. Crit Care.

[4] Peter JV, Moran JL, Philips-Hughes J, Warn D (2002). Noninvasive ventilation in acute respiratory failure--a
meta-analysis update. Crit Care Med.

[5] José A, Oliveira LR, Dias EC, Fuin DB, Leite LG, Guerra G de S (2006). Noninvasive positive pressure ventilation in patients with acute
respiratory failure after tracheal extubation. Rev Bras Ter Intensiva.

[6] Schettino G, Altobelli N, Kacmarek RM (2008). Noninvasive positive-pressure ventilation in acute respiratory
failure outside clinical trials: experience at the Massachusetts General
Hospital. Crit Care Med.

